# A new tribe of Elicinae Melichar, 1915 (Hemiptera, Tropiduchidae) with the description of a new genus and two new species from southern China

**DOI:** 10.3897/zookeys.1277.183491

**Published:** 2026-04-15

**Authors:** Huan Zhou, Thierry Bourgoin, Xiang-Sheng Chen, Jian-Kun Long, Zhi-Min Chang

**Affiliations:** 1 Institute of Entomology, College of Agriculture, Guizhou University, Guiyang, Guizhou, 550025, China Institut de Systématique, Evolution, Biodiversité, ISYEB-UMR 7205 MNHN-CNRS-Sorbonne Université-EPHE-University Antilles, Muséum National d’Histoire Naturelle Paris France; 2 The Provincial Special Key Laboratory for Development and Utilization of Insect Resources, Guizhou University, Guiyang, Guizhou, 550025, China College of Agriculture, Guizhou University Guiyang China; 3 Guizhou Key Laboratory of Agricultural Biosecurity, Guizhou University, Guiyang 550025, China Guizhou Key Laboratory of Agricultural Biosecurity, Guizhou University Guiyang China; 4 Institut de Systématique, Evolution, Biodiversité, ISYEB-UMR 7205 MNHN-CNRS-Sorbonne Université-EPHE-University Antilles, Muséum National d’Histoire Naturelle, CP 50, 57 rue Cuvier, 75005 Paris, France The Provincial Special Key Laboratory for Development and Utilization of Insect Resources, Guizhou University Guiyang China

**Keywords:** Morphology, planthopper, taxonomy

## Abstract

A new tribe, Elibucini**trib. nov**., is established in the subfamily Elicinae Melichar, 1915 (Hemiptera, Tropiduchidae) to accommodate the new genus *Elibuca* Zhou & Chang, **gen. nov**. The tribe is diagnosed by a distinctive combination of characters, including steeply tectiform forewings with well-developed nodal and subapical lines, hind tibiae lacking lateral spines, an elongate phallobase reaching or approaching the apex of the aedeagus, and rounded gonoplacs lacking marginal teeth. Two new species are described from southern China, and illustrated: *Elibuca clavata* Zhou & Chang, **sp. nov**. from Hainan, designated as the type species, and *E. lobata* Zhou & Chang, **sp. nov**. (Guizhou and Sichuan). Keys to the tribes of Elicinae and to the species of *Elibuca* are provided.

## Introduction

The family Tropiduchidae Stål, 1866 is a relatively moderately sized lineage of fulgoromorph planthoppers, currently including 689 species in 198 genera worldwide (Bourgoin 2025). Its global distribution spans both the northern and southern temperate zones, but the family is predominantly subtropical in the Northern Hemisphere, with a trimodal longitudinal pattern and a marked concentration of species in Asia (Bourgoin 2025). Tropiduchids inhabit a broad range of environments, from rainforests and macchia to semidesert biotopes, and feed primarily on shrubs, trees, and herbaceous plants. They are sap-sucking phytophagous insects, with recorded host associations concentrated principally with plants in the orders Ericales (9.2%) and Lamiales (9.2%), followed by Rosales, Poales, Gentianales, and Arecales (each around 8% of documented records) (Bourgoin 2025). Several species are known agricultural or forestry pests (Fennah 1982; Wang et al. 2016), such as *Kallitaxila sinica* on tallow trees, *Tambinia bambusana* on bamboo, and *Ommatissus lybicus* on date palm (Chang and Chen 2012; Bagheri et al. 2018; Ding et al. 2018).

Following Fennah’s (1982) global revision, which recognized 15 extant tribes, the higher classification of Tropiduchidae has been substantially restructured over the last two decades. A series of contributions (Gnezdilov 2007, 2013; Szwedo and Stroiński 2010, 2013, 2017; Gnezdilov et al. 2016; Wang et al. 2016; Stroiński et al. 2022) expanded the system into two subfamilies: Tropiduchinae Stål, 1866 and Elicinae Melichar, 1915, and increased tribal diversity to 26 tribes.

Tropiduchinae represent the “typical” tropiduchids, characterized by a distinctly flattened or shallowly tectiform body, elongated female genitalia, first valvula bearing a comb-like row of teeth, and a long gonoplac with marginal denticles (Fennah 1982; Gnezdilov et al. 2016). The subfamily currently includes 17 extant tribes and two fossil tribes (Bourgoin 2025).

The subfamily Elicinae was reinstated within Tropiduchidae by Gnezdilov (2013), who transferred Elicini Melichar, 1915 from Nogodinidae to Tropiduchidae, uniting it with Gaetuliini of Fennah (1978), and simultaneously establishing Parathisciini Gnezdilov, 2013. A further tribe, Bucini Gnezdilov, Bartlett & Bourgoin, 2016, was later added, along with an updated checklist including fossil taxa (Gnezdilov et al. 2016). Subsequently, the fossil tribe Gedanotropidini Szwedo & Stroiński, 2017 and the extant Laberiini Stroiński, Bourgoin & Szwedo, 2022 were described (Szwedo and Stroiński 2017; Stroiński et al. 2022), the later with comprehensive revision of the historical classification of the family and Elicini Melichar, 1915 (Stroiński et al. 2022).

As non-typical tropiduchids, Elicinae differ from Tropiduchinae by their more steeply tectiform body shape and by their issid-like female genitalia: rounded structures, triangular first valvulae with well-developed anterior connective laminae, and a gonoplac lacking marginal teeth or bearing only minute denticles (Gnezdilov 2013, 2016). Based on recent global revisions, the subfamily comprises four extant tribes: Bucini Gnezdilov, Bartlett & Bourgoin, 2016 (South America), Parathisciini Gnezdilov, 2013 (Africa), Laberiini Stroiński, Bourgoin & Szwedo, 2022 (Madagascar), Elicini Melichar, 1915 (cosmopolitan), and three fossil tribes: Austrini Szwedo & Stroiński, 2010, Patollini Szwedo & Stroiński, 2013, Gedanotropidini Szwedo & Stroiński, 2017 (all from Eastern Europe).

In China, Elicinae are represented only by the tribe Elicini, including two genera: *Indogaetulia* Schmidt, 1919 and *Connelicita* Wang & Bourgoin, 2015 (Chou and Lu 1977; Wang et al. 2015). No Chinese representatives of the other three extant tribes have been documented so far, suggesting that the Chinese fauna remains insufficiently explored and that additional undescribed taxa are likely to occur.

Recent fieldwork by our team yielded specimens of two distinctive species that show superficial similarity to members of Bucini, yet do not conform to the diagnostic characters of that tribe or of any other established tribe of Elicinae. Their unique morphological traits allow the recognition of a new genus including two new species, which are described and illustrated and support the formal establishment of a new tribe in China within Elicinae.

## Materials and methods

The external morphology was examined under a stereomicroscope. Body measurements were taken with a Nikon SMZ25 digital imaging system and are provided in millimetres (mm). Habitus photographs were captured using a Canon 5D Mark IV digital camera with an MP-E 65 mm f/2.8 1–5× macro lens and a Godox MF12 flash as the light source. Image stacks were processed with Zerene Stacker v. 1.04. Detailed photographs of selected body parts were obtained using a Keyence VHX-6000 system.

Abdomens of the examined specimens were removed and macerated in 10% KOH solution overnight, rinsed in distilled water, and then transferred into glycerine for examination. Genitalia were observed and illustrated under a Leica MZ 12.5 stereomicroscope. Illustrations were scanned using a CanonScan LiDE 200 and, together with photographs, arranged and labelled in Adobe Photoshop v. 23.2.1.

General external morphological terminologies follows Bourgoin and Huang (1990) for male genitalia and Bourgoin (1993) for female genitalia, Bourgoin et al. (2015) for tegmina and wing venation, as standardized and consistently applied in all recent tropiduchid studies including Gnezdilov (2002, 2013), Gnezdilov et al. (2014, 2016), Chang et al. (2017), and Song et al. (2024). The type specimens are deposited in the Institute of Entomology, Guizhou University, Guiyang, China (**GUGC**).

## Results

### Family Tropiduchidae Stål, 1866

**Subfamily Elicinae Melichar, 1915**

#### Tribe Elibucini

Taxon classificationAnimaliaHemipteraTropiduchidae

Zhou & Chang trib. nov.

792A4FF0-22A1-510C-8762-BC1E33100BB6

https://zoobank.org/3FB18689-3AA5-4B08-9D11-B01A5911A7E5

##### Type genus.

*Elibuca* Zhou & Chang, gen. nov.

##### Diagnosis.

**Figure 1.**
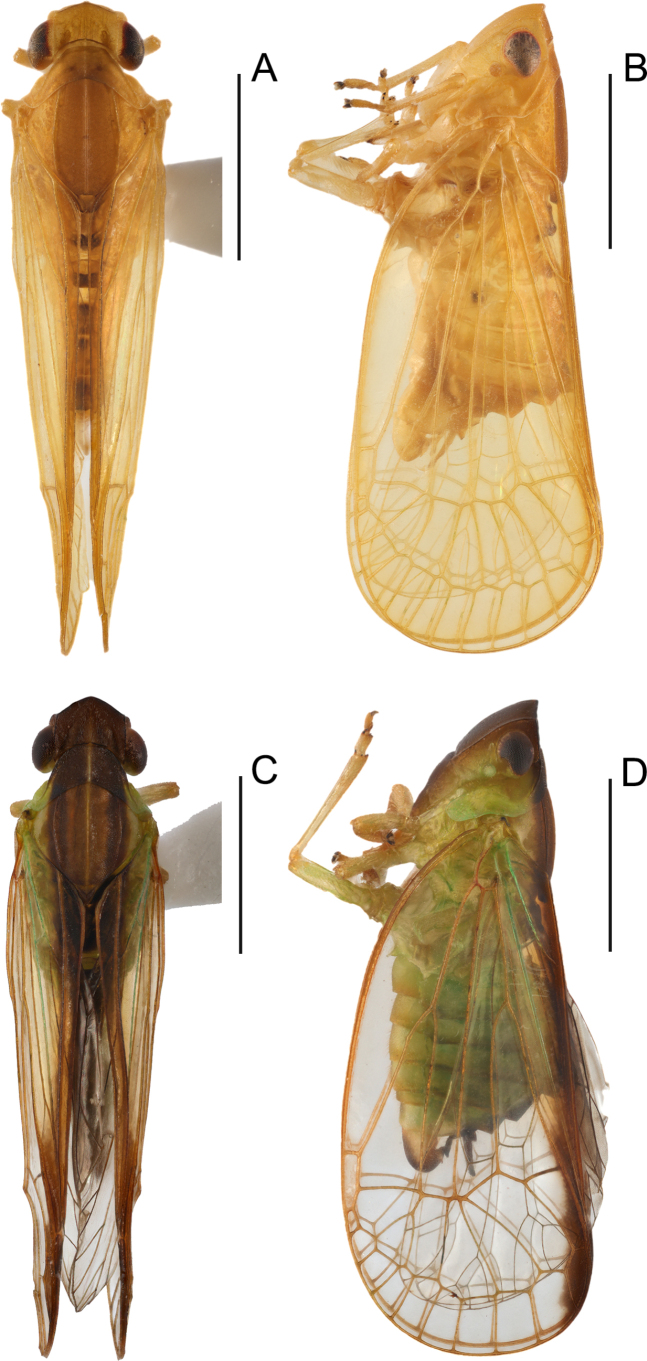
Male habitus of *Elibuca* species. **A, B**. *Elibuca clavata* Zhou & Chang, sp. nov.; **C, D**. *Elibuca lobata* Zhou & Chang, sp. nov.; **A, C**. Dorsal view; **B, D**. Lateral view. Scale bars: 1 mm.

**Figure 2.**
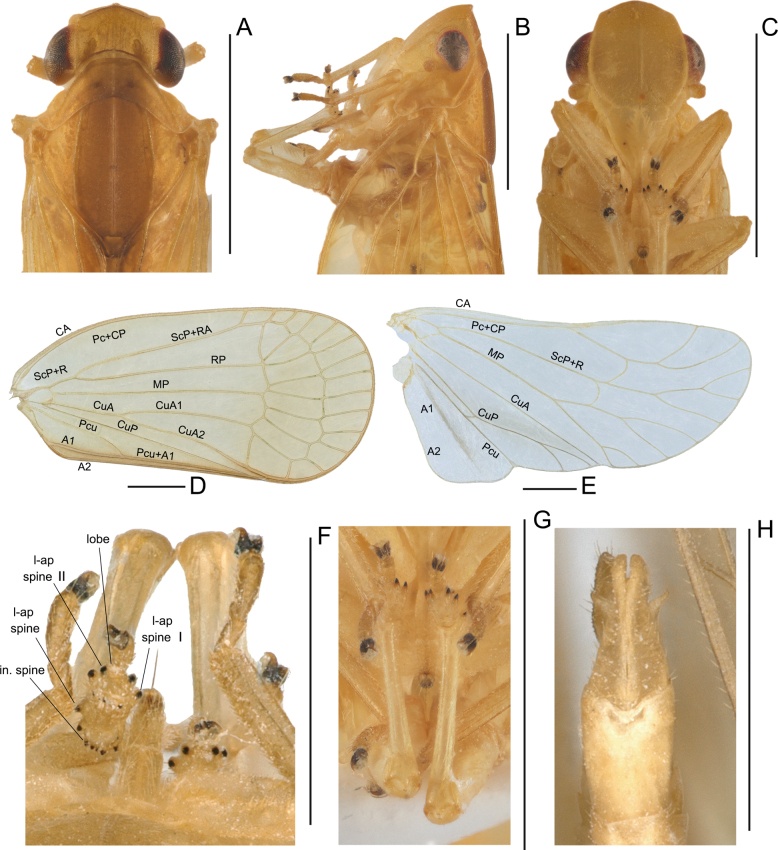
*Elibuca clavata* Zhou & Chang, sp. nov. male. **A**. Head and thorax, dorsal view; **B**. Head and thorax, left view; **C**. Head and thorax, ventral view; **D**. Forewing; **E**. Hind wing; **F**. Apex of hind tibiae and metatarsomeres, dorsal view; **G**. Hind tibiae and metatarsus, ventral view; **H**. Male genitalia, ventral view. Abbreviations: in. spine = apical intermediate spines of tibiae; l-ap spine = latero-apical spines of tibiae; l-ap spine I = latero-apical spines of 1^st^ metatarsomere; l-ap spine II = latero-apical spines of 2^nd^ metatarsomere; lobe = ventral lobe of second metatarsomere. Scale bars: 1 mm.

**Figure 3.**
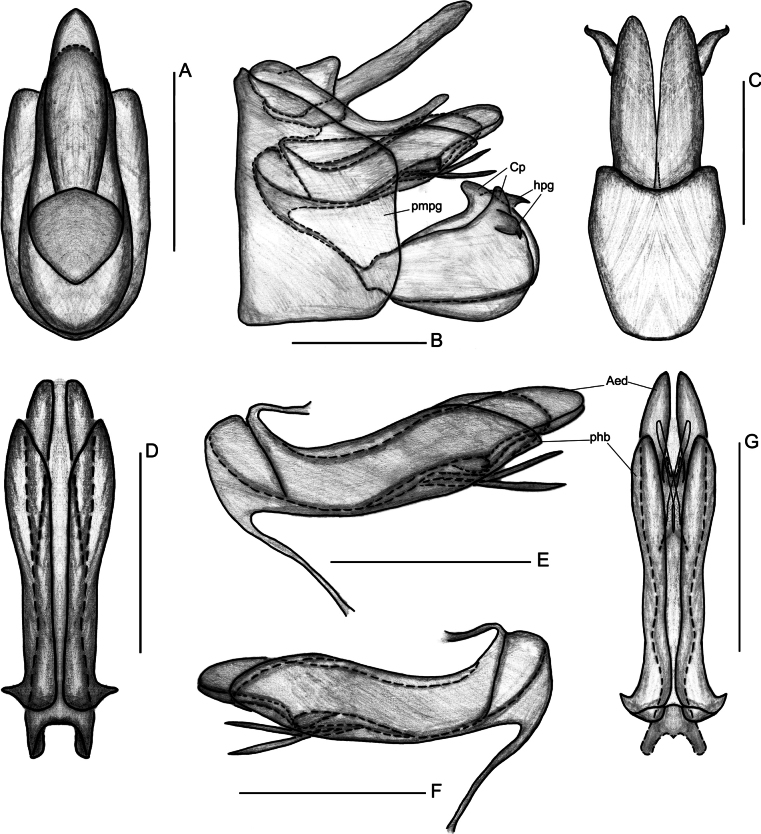
*Elibuca clavata* Zhou & Chang, sp. nov. male. **A**. Pygofer and anal tube, dorsal view; **B**. Male genitalia, left view; **C**. Pygofer and gonostyli, ventral view; **D**. Apex of aedeagus and phallobase, dorsal view; **E**. Aedeagus, left view; **F**. Aedeagus, right view; **G**. Apex of aedeagus and phallobase, ventral view. Abbreviations: Aed = aedeagus, Cp = capitulum of gonostylus, hpg = hook-like process of gonostylus, phb = phallobase, pmpg = posterior margin of pygofer. Scale bars: 0.5 mm.

The new tribe is readily distinguished from Elicini (Gnezdilov 2013, 2016; Wang et al. 2015) in the following combination of characters: 1) forewings extending well beyond the abdomen, the costal area lacking crossveins (Figs 1B, 2D) (vs forewings short or elongate, with crossveins in the costal area in Elicini); 2) hind tibiae lacking lateral teeth (Fig. 2G) (vs hind tibiae with more than two lateral teeth); 3) phallobase markedly elongate, reaching or approaching the apex of the aedeagus (Fig. 3E) (vs short and not reaching the apex).

**Figure 4.**
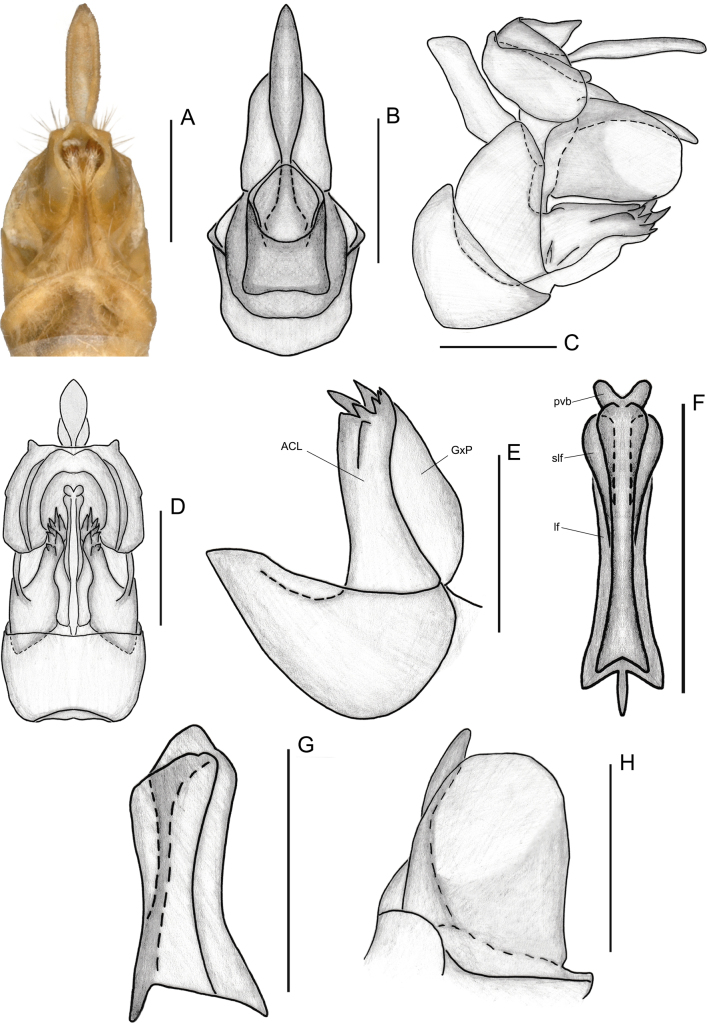
Female genitalia of *Elibuca clavata* Zhou & Chang, sp. nov. **A**. Ventral view; **B**. Dorsal view; **C**. Lateral view; **D**. Posterior view; **E**. Gonapophyses VIII, lateral view; **F**. Gonapophyses IX, ventral view; **G**. Gonapophysis IX, lateral view; **H**. Gonoplac, lateral view. Abbreviations: ACL = anterior connective lamina of gonapophysis VIII, GxP = endogonocoxal process, lf = lateral field of posterior connective lamina of gonapophysis IX, pvb = posterior ventral lobe, slf = sublateral field of posterior connective lamina of gonapophysis IX. Scale bars: 0.5 mm.

From Laberiini (Stroiński et al. 2022), the new tribe differs in possessing: 1) forewings steeply tectiform and without reticulate venation (Figs 1B, 2D) (vs weakly tectiform, broad, and reticulate in Laberiini); 2) hind tibiae without lateral teeth (Fig. 2G) (vs hind tibiae bearing 5–7 lateral spines); 3) anterior connective lamina of gonapophyses VIII with two well-defined rows of apical teeth (Fig. 4E) (vs a single row of teeth along the apical and lateral margins).

From Parathisciini (Gnezdilov 2013), the new tribe may be separated by:1) forewing transparent with simple venation (Fig. 1B) (vs forewing opaque, with distinct reticulate venation); 2) phallobase elongate and reaching or approaching the apex of the aedeagus (Fig. 3E) (vs phallobase short, not reaching the apex).

Finally, the new tribe shows some superficial similarity to Bucini but differs by: 1) forewing with a distinct nodal line and subapical line forming well-delimited subapical and apical cells, and without transverse veinlets *cup*–(*pcu+a1*) distally on the clavus (Fig. 2D) (vs forewing lacking nodal and subapical lines); 2) hind tibiae without lateral spine (Fig. 2G) (vs hind tibiae with one lateral spine); 3) gonostyli with a hooked process near the base of the capitular neck, capitulum lacking a plate-shaped tooth (Fig. 3B) (vs gonostyli without a hooked process and capitulum bearing a plate-shaped tooth); 4) posterior connective lamina of gonapophyses IX (second valvula) rod-like and slender (Fig. 4F) (vs second valvula triangular with a more developed posterior connective lamina).

##### Description.

Head (Fig. 2A) projected in front of eyes, narrower than thorax. Vertex broad or long. Frons with median carina (Fig. 2C). Rostrum (Fig. 2C) short, just reaching or exceeding the middle coxae. Pronotum and mesonotum both tricarinate (Fig. 2A). Forewings (Figs 1B, 2D) translucent, well surpassing the abdomen, laterally compressed, subparallel to body; with costal area lacking transverse veins, ScP+R and CuA forked before fusion of Pcu+A1, MP simple to nodal line level, with distinct nodal line and subapical line, clavus with weak tranverse veins between CuP and Pcu. Hind wings (Fig. 2E) inconspicuously trilobed, nocked near apex of CuA and Pcu; CuA single, with a transverse veins between *cup*–*pcu*. Hind tibiae (Fig. 2F, G) without lateral spine, with asymmetrical apical spinulation; 2^nd^ metatarsus with symmetrical apical spinulation. Hind wings with a transverse crossvein on each side of ScP+R; A1 not reaching posterior margin (Fig. 2E). Male genitalia with gonostyli hooked process near base of neck in capitulum (Fig. 3B, C); phallobase (Fig. 3E, F) slightly recurved, well-developed, approaching or reaching the apex of the aedeagus. Female genitalia (Fig. 4A, D) rounded, anal style very long (Fig. 4C), anterior connective lamina of gonapophysis VIII (Fig. 4D) symmetrical; posterior connective lamina of gonapophysis IX (Fig. 4F) symmetrical and slender, rod-liked; gonoplacs (Fig. 4C, H) rounded, without marginal teeth.

### Key to extant tribes of the subfamily Elicinae Melichar, 1915

**Table:** 

1	Forewings opaque, with notably prominent reticulate veins in entire surface	** Parathisciini **
–	Forewings translucent, without significant prominent reticulate veins in entire surface	**2**
2	Forewings short, reduced, or well developed with many transverse veins in costal area	**3**
–	Forewings well developed, without transverse veins in costal area	**4**
3	In dorsal view, lateral margin of gonapophysis protruded with a short, tooth-like process	** Elicini **
–	In dorsal view, lateral margin of gonapophysis bearing a long, digitate process	** Laberiini **
4	Forewing with CuA single, with transverse vein *cup-*(*pcu+a1*) in clavus; hind tibiae with a single lateral spine; gonostyli without hooked process near base of neck in capitulum	** Bucini **
–	Forewing with CuA forked, without transverse vein *cup*-(*pcu+a1*) in clavus, with transverse veins between CuP and Pcu; hind tibiae without lateral spine; gonostyli with hooked process near base of neck in capitulum	**Elibucini trib. nov**.

#### Genus Elibuca

Taxon classificationAnimaliaHemipteraTropiduchidae

Zhou & Chang gen. nov.

61EFBA41-E043-54C0-828F-9E993C4CE486

https://zoobank.org/1489B3A6-AEF2-4242-9B21-17D49A138081

##### Type species.

*Elibuca clavata* Zhou & Chang, sp. nov., here designated.

##### Etymology.

Arbitrary combination of the generic names *Elica* Walker, 1857 and *Buca* Walker, 1857, referring to the tribes Elicini Melichar, 1915, and Bucini Gnezdilov, Bartlett & Bourgoin, 2016, to which these genera belong. The name is formed for convenience and euphony only and does not imply any phylogenetic relationship with either tribe at this stage. The gender is feminine.

##### Description.

Body medium-sized (body length: 7.3–7.7 mm).

**Figure 5.**
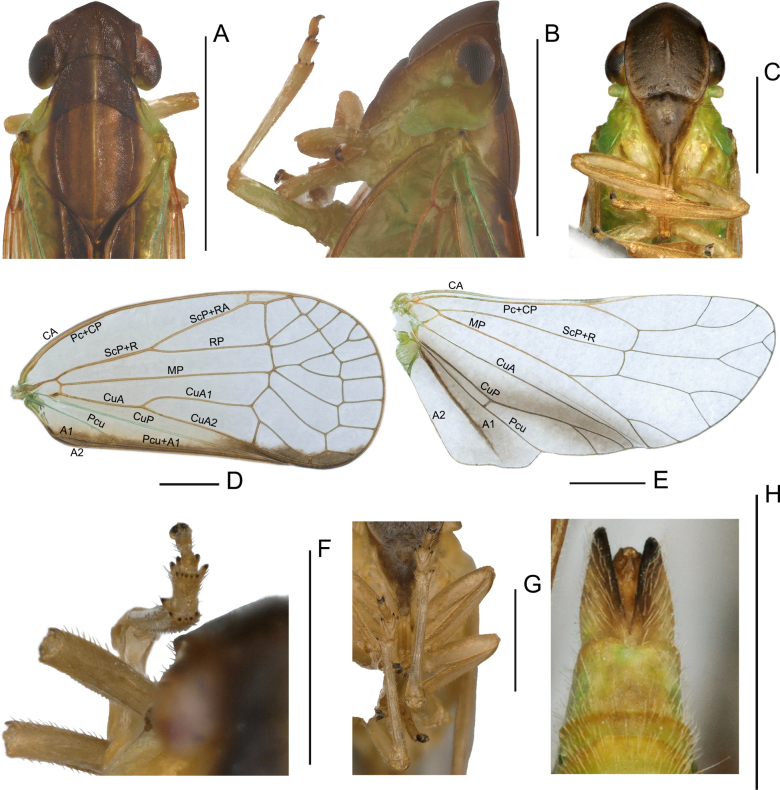
*Elibuca lobata* Zhou & Chang, sp. nov. male. **A**. Head and thorax, dorsal view; **B**. Head and thorax, left view; **C**. Head and thorax, ventral view; **D**. Forewing; **E**. Hind wing; **F**. Apex of metatibia and metatarsus, dorsal view; **G**. Apex of metatibia and metatarsomeres, ventral view; **H**. Male genitalia, ventral view. Scale bars: 1 mm.

***Head and thorax***. Head including eyes distinctly narrower than pronotum (Figs 2A, 5A). Vertex (Figs 2A, 5A) irregularly quadrangular, shorter in middle than width in dorsal view, disc of vertex depressed, with or without median carina; anterior margin angularly produced before eyes; posterior margin concavely arched; lateral margins parallel. Frons (Figs 2C, 5C) irregularly hexagonal, longer than maximum width, with median carinate, maximum width above level of antenna; frontoclypeal suture nearly straight. Clypeus (Figs 2C, 5C) triangular, length distinctly exceeding width, with median carina but without lateral carina. Rostrum (Figs 2C, 5C) short, just reaching middle coxae. Pronotum (Figs 2A, 5A) tricarinate, with stout median carina and lateral carinae present; posterior margin concavely arched, wider than long. Mesonotum (Figs 2A, 5A) tricarinate; median carina straight, reaching to mesoscutellum; lateral carinae curving towards median carina; rhombic, with the length and width nearly subequal. Forewing (Figs 2D, 5D) translucent and oval, more than 2.00 times as long as maximum width; anterior and posterior margins nearly parallel; basal cell medium-sized; costal area present without crossveins; ScP+R vein forked, ScP+RA bifurate before nodal line; MP simple, reaching to nodal line; CuA bifurcated near basal region; CuP simple, reaching near apical margin; Pcu and A1 uniting before clavus; clavus with 1–2 transverse veins between CuP and Pcu, without transverse vein *cup*-(*pcu+a1*) in distal position, with distinct nodal line and subapical line, forming 8–9 subapical cells and 11–13 apical cells. Hind wings (Figs 2E, 5E) triangular, inconspicuously trilobed; ScP+R forked near apical margin; MP forked into branches, with a partially visible vein extending from the wing margin and abbreviating in the medial area near CuA; CuA single; CuP forked near apical margin, with a transverse vein between CuP and Pcu; A1 single, evanescing before reaching external margin. Hind tibiae (Figs 2F, 2G, 5F, 5G) without lateral spine, with 1 latero-apical spine and row of 5–6 intermediate spines; first metatarsomere with 2 latero-apical spines and row of 5–6 intermediate spines between them; 2^nd^ metatarsus with two symmetrical apical spines and a median lobe between them.

**Figure 6.**
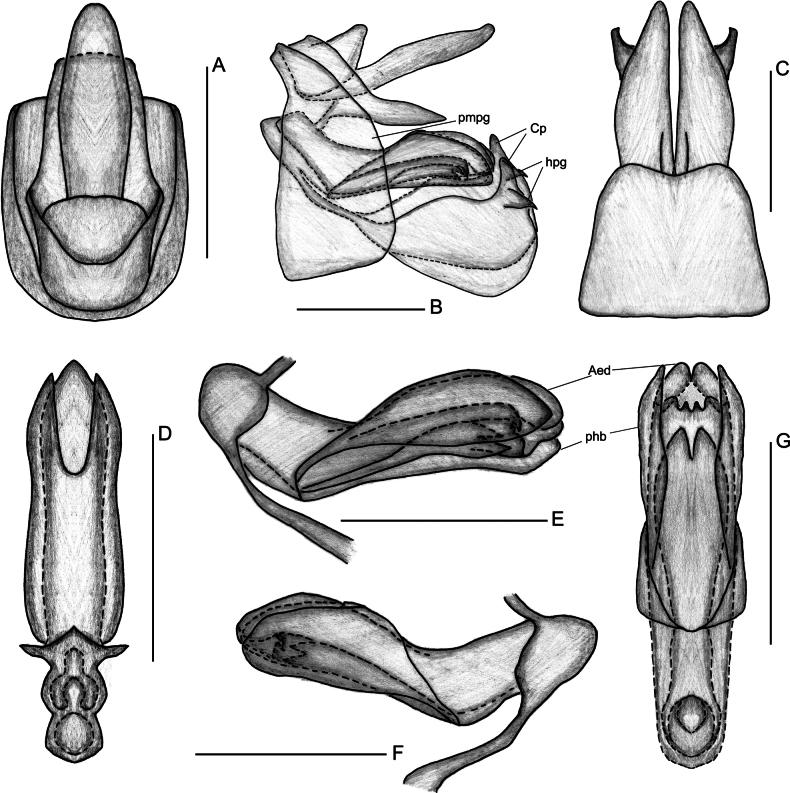
*Elibuca lobata* Zhou & Chang, sp. nov. male. **A**. Pygofer and anal tube, dorsal view; **B**. Male genitalia, left view; **C**. Pygofer and gonostyli, ventral view; **D**. Apex of aedeagus and phallobase, dorsal view; **E**. Aedeagus, left view; **F**. Aedeagus, right view; **G**. Apex of aedeagus and phallobase, ventral view. Abbreviations: Aed = aedeagus, Cp = capitulum of gonostylus, hpg = hook-like process of gonostylus, phb = phallobase, pmpg = posterior margin of pygofer. Scale bars: 0.5 mm.

***Male genitalia***. Anal tube (Figs 3A, 6A) symmetrical, oblong, longer in middle than base; anal style elongate, far exceeding apex of the anal tube in dorsal view. Pygofer (Figs 3B, 6B) bilaterally symmetrical, irregularly trapezoidal in lateral view, concave on posterior margin in ventral view. Gonostyli (Figs 3B, 6B) symmetrical, irregularly elliptic, fused at base in ventral view, with a hooked process near base of neck in capitulum. Capitulum of gonostyli long, apically narrowing. Phallobase (Figs 3E, 6E) symmetrical, tube-liked, basally fused, extending beyond middle length of aedeagus or nearly to aedeagal apex. Aedeagus (Figs 3D–G, 6D–G) bilaterally symmetrical, tubular, medially connected by thin membranous layer in dorsal view, with a median depression bearing paired processes in ventral view.

**Figure 7.**
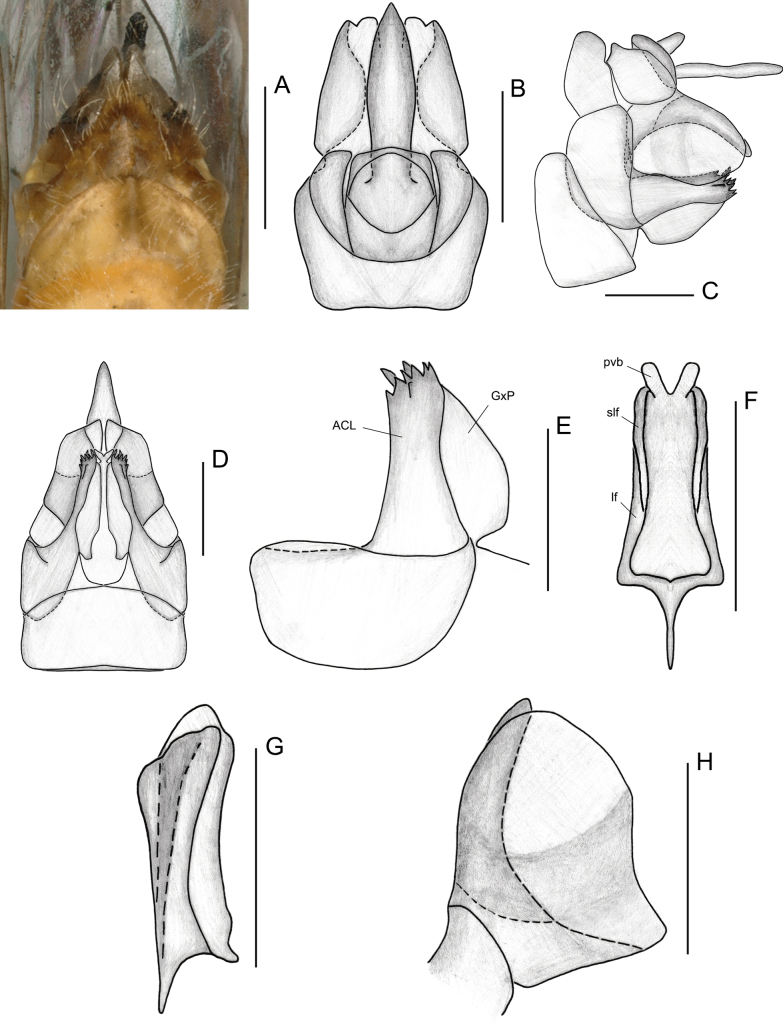
Female genitalia of *Elibuca lobata* Zhou & Chang, sp. nov. **A, D**. Ventral view; **B**. Dorsal view; **C**. Lateral view; **E**. Gonapophysis VIII, lateral view; **F**. Gonapophyses IX, ventral view; **G**. Gonapophysis IX, lateral view; **H**. Gonoplac, lateral view. Abbreviations: ACL = anterior connective lamina of gonapophysis VIII, GxP = endogonocoxal process, lf = lateral field of posterior connective lamina of gonapophysis IX, pvb = posterior ventral lobe, slf = sublateral field of posterior connective lamina of gonapophysis IX. Scale bars: 0.5 mm.

***Female genitalia***. Genitalia bilaterally symmetrical (Figs 4A, 7A). Anal tube (Figs 4B, 7B) symmetrical, very short; anal style conspicuously long, exceeding apex of anal tube. Gonapophyses VIII (first valvula) (Figs 4E, 7E) broad, slightly longer than wide; endogonocoxal lobe not obvious. Anterior connective lamina of gonapophyses VIII broad, sclerotized at apex, with two distinct rows of teeth on apical margin; endogonocoxal process developed. Gonapophyses IX (second valvula) (Figs 4F, 7F) bilaterally symmetrical. Posterior connective lamina of Gonapophyses IX somewhat sclerotized, rod-like, and slender, without obvious median field. Gonoplacs (third valvula) (Figs 4H, 7H) broad, membranous, subcircular, without marginal teeth.

##### Host plant.

Unknown.

##### Distribution.

China (Guizhou, Hainan, Sichuan).

### Key to species of *Elibuca* Zhou & Chang, gen. nov.

**Table:** 

1	Anterior margin of vertex nearly straight; forewing without irregular, dark bands near inner margin; aedeagus with 2 clavate processes (Figs 1A, 2D, 3G)	***Elibuca clavata* sp. nov**.
–	Anterior margin of vertex not nearly straight; forewing with irregular dark bands near the inner margin; aedeagus with a bilobate process (Figs 1C, 5D, 6G)	***Elibuca lobata* sp. nov**.

#### Elibuca clavata

Taxon classificationAnimaliaHemipteraTropiduchidae

Zhou & Chang sp. nov.

62740C2B-FC71-5DA9-B24A-1DC0713A3FDC

https://zoobank.org/3309E3C0-E26C-41B0-9223-CC9BD920AC62

Figs 1A, 1B, 2A–H, 3A–G, 4A–H

##### Type material.

***Holotype***: • ♂, China Hainan Province, Lingshui Li and Miao Autonomous County, Diaoluoshan National Forest Park (18°39'57"N, 109°55'59"E), 5 May 2021, N. Gong leg.; IEGU 181111. ***Paratypes***: • 2♂♂, same data as holotype, 30 Apr. 2021, L. Zhang leg.; • 2♀♀, same data as holotype, 30 Apr. 2021, Y.-J. Sui leg.; IEGU 181112, IEGU 181113, IEGU 181121, IEGU 181122.

##### Description.

***Measurements***. Body length (from apex of vertex to tip of fore wings): male 7.5–7.7 mm (*N* = 3), female 7.3–7.5 mm (*N* = 2).

***Colouration***. General colour yellowish green (Fig. 1A). Vertex, pronotum and mesonotum tan to dark brown, along the median carina infuscate (Fig. 1A). Forewings transparent, pale yellow, slightly darkened near the inner margin (Fig. 2D). Hind wings transparent, pale-brown bands surrounding CuA, CuP, and A1 veins (Fig. 2E). Abdomen yellow (Fig. 1A).

***Head and thorax***. Vertex (Fig. 2A) broader than long in the middle line (2.02: 1.00); disc distinctly depressed with 2 dark marks, with faint median carina, reaching to base 1/3 of vertex. Frons (Fig. 2C) longer than the maximum width (1.20: 1). Clypeus (Fig. 2C) triangular, with a faint median carina. Pronotum (Fig. 2A) anterior margin nearly straight, posterior margin concavely arched; lateral carinae converged with median carina; width wider than length at middle (3.06: 1.00) and length shorter than vertex at middle (1.00: 1.10); sunken pits present between median carina and lateral carinae. Mesonotum (Fig. 2A) with width and length at middle almost equal (1.01: 1.00) and longer than length of vertex and pronotum together (2.61: 1.00). Forewing (Fig. 2D) 2.21 times longer than its maximum width, with outer margin relatively broad. ScP+R vein forked near the base 1/6; CuA forked two branches in basal 1/4 before Pcu and A1 unite, Pcu and A1 uniting near middle of clavus, with 8 subapical cells and 12 apical cells. Hind wings (Fig. 2E) with venation relatively simple; venation as Fig. 2E.

***Male genitalia***. Anal tube (Fig. 3A, B) irregularly elliptical, with the apical margin distinctly convex in dorsal view. Anal style very long, exceeding 1/3 of apex of anal tube. Pygofer (Fig. 3B) irregularly trapezoidal in lateral view, distinctly broad; dorsal margin inclining towards ventro-posterior margin; posterior margin with two weak projections in lateral view; in ventral view (Fig. 3C), posterior margin obviously concave. Gonostyli (Fig. 3B) irregularly elliptic, narrower at base and expanding at apex, distinctly longer than wide, with a stout, hooked process near base of capitulum, pointing to ventral margin; neck of capitulum stout. Phallobase (Fig. 3D–G) tubular; apex reaching to 2/3 of aedeagus in lateral view; apical part medially divided in dorsal view, with a deep median incision in ventral view. Aedeagus (Fig. 3E–G) slightly dorsally curved, enlarged and rounded at apex, with two clavate processes in ventral view, connected to left and right sides at base of median depression, and before with two slender clavate processes projecting and pointing to caudad.

***Female genitalia***. Anal tube (Fig. 4B) short, shorter in middle than wide (1.00:3.43); anal style long and thin, 6.86 times longer in middle than anal tube, with base narrower. Gonapophyses VIII (Fig. 4C, E) broad, slightly longer than wide; endogomocoxal process membranaceous. Anterior connective lamina of gonapophyses VIII (Fig. 4C, E) of moderate width, with a proximal row with 5 smaller teeth and a distal row of 2 larger teeth on apical margin. Gonapophyses IX (second valvula) (Fig. 4F) elongate and slender; posterior ventral lobes and lateral and sublateral field of posterior connective lamina extended and slimmer. Posterior margin of sternite VII (Fig. 4D) slightly arched; anterior margin of sternite VII almost straight, with a very subtle concavity.

##### Host plant.

Unknown.

##### Etymology.

The species name is derived from the Latin word *clavata*, referring to the two clavate processes in the aedeagus.

##### Distribution.

Known only from the type locality.

#### Elibuca lobata

Taxon classificationAnimaliaHemipteraTropiduchidae

Zhou & Chang sp. nov.

C9CA1C8B-BBFA-5269-AD60-4DEA73820B27

https://zoobank.org/05B9ABF2-0EC5-438F-AC46-215C12D6D6E0

Figs 1C, 1D, 5A–H, 6A–G, 7A–H

##### Type material.

***Holotype***: • ♂, China Sichuan Province, Ya’an City, Yingjing County, Longgou Town, Mutiyan Rock (29°40'57"N, 102°51'37"E), 30 Jul. 2022, S.-S. Lv leg.; IEGU 181211.

***Paratypes***: •♂, China Guizhou Province, Zunyi City, Suiyang County, Kuankuoshuishui Nature Reserve 11 Jul. 2023, L.-L. Shan leg.; •♀, same date as the male: IEGU 181212, IEGU 181221.

##### Description.

***Measurements***. Body length (from apex of vertex to tip of fore wings): male 7.5–7.7 mm (*N* = 2), female 7.7 mm (*N* = 1).

***Colouration***. General colour green to brown (Fig. 1C). Vertex and pronotum sable; mesonotum brown, light brown around the median carina (Fig. 5A). Forewings transparent, pale yellow, becoming dark brown near inner margin (Fig. 5D). Hind wings transparent; dark-brown bands surround region from veins CuA to A1 (Fig. 5E). The abdomen green-brown (Fig. 1C).

***Head and thorax***. Vertex (Fig. 5A) wider than long in mid-line (1.55: 1.00); disc depressed, with stout median carina, reaching to base 3/4 of vertex. Frons (Fig. 5C) longer than maximum width (1.20: 1.00). Clypeus (Fig. 5C) triangular; postclypeus without median carina; anteclypeus with a distinct median carina. Pronotum (Fig. 5A) trapezoidal; anterior margin convexly arched; posterior margin with an obtuse indented angle; lateral carinae converged with median carina, wider than long at middle (2.30: 1.00), and shorter than vertex at middle (1.00: 1.25), with sunken pits between median carina and lateral carinae. Mesonotum (Fig. 5A) almost as long as wide in middle (1.01: 1), and longer than combined length of vertex and pronotum (1.55: 1). Forewing (Fig. 5D) 2.11 times longer than widest breadth, with irregular, obviously dark bands near inner margin; ScP+R vein forked near base 1/3; CuA forked 2 branches in basal 1/3 after where Pcu and A1 unite; Pcu and A1 uniting before middle of clavus, with 8 subapical cells and 9–10 apical cells. Hind wings with venation relatively simple, as in Fig. 5E.

***Male genitalia***. Anal tube (Fig. 6A) irregularly quadrangular; apical margin straight in dorsal view. Anal style long, exceeding the 1/5 of the apex of the anal tube. Pygofer (Fig. 6B) irregularly trapezoidal in lateral view, relative narrow, dorsal margin obviously inclining towards ventro-posterior margin, posterior margin relatively straight and parallel to anterior margin; in ventral view, posterior margin slightly concave. Gonostyli (Fig. 6B) irregularly subquadrate, narrower at base and expanding at apex, slightly longer than wide, with a stout, hooked process near base of capitulum, pointing to posterior margin; neck of capitulum stout. Phallobase (Fig. 6D–G) oval, encapsulating apical part of aedeagus in lateral view; apical part obtuse in dorsal view, with a shallow incision in ventral margin. Aedeagus (Fig. 6E–G) slightly dorsally curved, medially narrow in ventral view, medially concave; basal margin with 2 semicircular processes and 2 cornute processes, with a bilobate process before 4 processes.

***Female genitalia***. Anal tube short, shorter in middle than width (1.00: 3.17); anal style (Fig. 7B, C) relatively short and robust, longer at middle than anal tube 5.67 times. Gonapophyses VIII (first valvula) (Fig. 7C, E) broader, longer than wide at middle; endogomocoxal process membranaceous. Anterior connective lamina of Gonapophyses VIII wider, with 2 distinct rows of teeth on apical margin of anterior connective lamina; first row with 6 large teeth and second row with 2 small teeth in lateral view. Gonapophyses IX (second valvula) (Fig. 7F) elongate, relatively wider; posterior ventral lobes and lateral and sublateral field of posterior connective lamina (Fig. 7F) extended and relatively broader. Posterior margin of sternite VII (Fig. 7D) straight; anterior margin of sternite VII slightly convex, forming a gentle arc.

##### Host plant.

Unknown.

##### Etymology.

The name of the new species is derived from the Latin word *lobata*, “having lobes”, referring to the bilobate process in the aedeagus.

##### Distribution.

Known only from the type locality and a single locatity in Guizhou.

## Discussion

Based on a combination of diagnostic characters, including steeply tectiform forewings, a rounded ovipositor, and gonoplacs lacking marginal teeth (Gnezdilov 2013), the new tribe Elibucini trib. nov. can be confidently assigned to the subfamily Elicinae.

However, the presence of distinct nodal and subapical lines in the forewings of Elibucini, characters that are absent in other Elicinae but typical of Tropiduchinae (Gnezdilov et al. 2016), indicates a mosaic of character states within the tribe.

Although clearly distinct at the tribal level, Elibucini shares certain morphological features with other Elicinae taxa. In particular, the flat, rounded gonoplacs lacking marginal teeth, combined with the presence of two apical rows of teeth on gonapophyses VIII in the female genitalia, are reminiscent of Parathisciini (Gnezdilov 2013). Additionally, the absence of a distal transverse vein *cup*–(*pcu+a1*) and the phallobase almost completely enclosing the aedeagus resemble conditions observed in Bucini (Gnezdilov et al. 2016).

This combination of characters suggests that Elibucini occupies a morphologically intermediate position between Parathisciini and Bucini. Nevertheless, such an interpretation remains hypothetical and should not be taken as evidence of a direct evolutionary transition between these tribes. Molecular phylogenetic data, currently unavailable for this lineage, will be required to test this hypothesis and to clarify the evolutionary relationships within Elicinae.

Furthermore, within this tribe, the A1 vein of the hind wings does not reach the posterior margin, and its posterior half is relatively indistinct—a condition similar to that observed in some species within the tribe Elicini (Wang et al. 2015), although rarely seen elsewhere in Tropiduchidae. Additionally, the presence of the cup–pcu crossvein, which has not been found in other tribe Tropiduchidae, is noteworthy. At present, hind wings venation is seldom employed in the taxonomic identification of Tropiduchidae. However, based on the findings presented in this study, it is recommended that hind wings venation characteristics—particularly the morphology of vein A1 and the presence or absence of the cup–pcu crossvein—be considered as supplementary diagnostic features in future taxonomic studies.

## Supplementary Material

XML Treatment for Elibucini

XML Treatment for Elibuca

XML Treatment for Elibuca clavata

XML Treatment for Elibuca lobata

## Additional information

### Use of AI

No use of AI was reported.

### Author ORCIDs

Huan Zhou https://orcid.org/0009-0000-6887-8221

Thierry Bourgoin https://orcid.org/0000-0001-9277-2478

Xiang-Sheng Chen https://orcid.org/0000-0001-9801-0343

Jian-Kun Long https://orcid.org/0000-0002-4576-9919

Zhi-Min Chang https://orcid.org/0000-0002-7247-9706

### Data availability

All of the data that support the findings of this study are available in the main text.
